# PEEK Composites as Self-Lubricating Bush Materials for Articulating Revolute Pin Joints

**DOI:** 10.3390/polym12030665

**Published:** 2020-03-17

**Authors:** Juanjuan Zhu, Fang Xie, R S Dwyer-Joyce

**Affiliations:** 1 The Leonardo Centre for Tribology, Department of Mechanical Engineering, University of Sheffield, Mappin Street, Sheffield S1 3JD, UK; r.dwyer-joyce@sheffield.ac.uk; 2 School of Mechanical & Automotive Engineering, Nanyang Institute of Technology, Nanyang 473004, China

**Keywords:** PEEK composites, reinforcements, self-lubricating bush, friction and wear, pin joints

## Abstract

In this study, bearing bushes made of polyetheretherketone (PEEK), 30 wt % carbon fibre reinforced PEEK, 30 wt % glass fibre reinforced PEEK, each 10 wt % of PTFE, graphite and carbon fibre modified PEEK were investigated on a purpose built pin joint test rig. The unlubricated friction and wear behaviour was assessed in sliding contact with a 300M shaft, subjected to a nominal pressure of 93 MPa, articulating sliding speed of 45 °/s. The worn surface and the subsurface layer were studied using optical profilometry and scanning electron microscopy (SEM). Due to thermal sensitivity of PEEK composites, friction energy and temperature rise were analysed for determining the friction and wear mechanism. The bush made of PTFE, graphite and carbon fibre (each 10 wt %) modified PEEK presented the best performance for friction coefficient, wear loss, friction energy and temperature rise. Current work demonstrated that reinforcement modified PEEK composite possesses desirable properties to perform as a load bearing bush in certain tribological applications.

## 1. Introduction

Compared with metals, polymers possess certain desired properties for engineering use, i.e., lightweight (low density), low cost, ease of manufacturing, self-lubricating and corrosion resistance [1,2,3,4]. They can provide significant weight savings while maintaining structural performance, and therefore offering improved fuel efficiency for aerospace and other transport applications. In addition, polymers are increasingly used in tribological applications, especially for harsh lubrication conditions, such as bearings, gears, piston rings and seals in aerospace machines and ocean engineering machines or other mechanical components used in high temperature and corrosive environment [5,6,7,8].

Among the speciality polymers, polyetheretherketone (PEEK) is one of the most promising engineering materials for tribological applications. Studies have been conducted on the friction and wear of pure PEEK in comparison with other polymers [9,10]. However, there are limitations of pure PEEK, such as low thermal stability, heat conductivity and dissipativity. In order to minimise these disadvantages and to further improve the friction and wear property, PEEK based composites have been tailored with variety of reinforcements, fillers and solid lubricants [11]. In the past twenty years, researchers have made great efforts to develop PEEK-based composites. Mechanical strength, friction and wear properties were studied for carbon fibre reinforced PEEK composites [12,13]. Sumer et al. [14] reported that the glass fibre in the composite improved friction and wear under dry sliding contact. Wang et al. reported that the composite with 7.5 wt % ZrO_2_ particles produced a low wear rate and friction coefficient through the block-on-ring tests (PEEK composite block against sliding steel ring) due to the formation of a thin, uniform and tenacious transfer film at the interface [15]. The influence of Polytetrafluoroethylene (PTFE) on the mechanical and tribological properties were studied by Zhang et al. [16] and Bijwe et al. [17].

Tribological behaviour of PEEK composites is also affected by the operating environment, i.e., gas, temperature, lubricant, load, etc. [18,19,20,21]. Theiler and Gradt evaluated the tribological behaviour of PEEK composites in air, vacuum and hydrogen environments [18] from pin-on-disc (PEEK composite pin against steel disc) contact. It was found that PEEK composites presented lower environmental sensitivity compared with pure PEEK [18]. Varying lubricants, i.e., water [6,14], sea water [1], mineral oil [5,21,22] were used in the study of friction and wear for PEEK composites. Zhang et al. observed enhanced lubricity under boundary and mixed lubrication regime for the PEEK composites reinforced with graphitic carbon nitride nanosheets when lubricated by PAO4 oil through the plate-on-ring (PEEK composite plate against sliding steel ring) tests [5].

Tribological characteristics of PEEK composites are highly dependent on the tribo-system. The friction energy dissipated in the sliding contact usually causes a consistent temperature rise in the two contacting bodies [7,23]. The temperature variation in service plays an important role in affecting the mechanical, physical and thermal properties, resulting in structural changes of polymer components. Most of the work relating to PEEK composites has been conducted in the lab using standard tribo-meters. For engineering use, some research has been carried out where PEEK composites form the tribological component, including ball bearings [8,24], thrust bearing [25], orthopaedic device [26] and crank shaft bush on the robot joint [27]. There is no work conducted towards journal bearing bushes made of PEEK composites. The aim of the current work is to investigate the tribological performance of PEEK composite used as bearing bushes through a purpose build pin joint test rig subjected to a contact pressure of 93 MPa without lubrication. A thorough assessment was conducted on tested bushes, including friction coefficient, bush wall deformation, wear rate, friction energy and temperature increase. The wear tracks and the subsurface layer were examined to assess the tribological behaviour of PEEK composites used as load bearing bush material.

## 2. Experimental Methods 

### 2.1. Specimen

**Figure 1:**
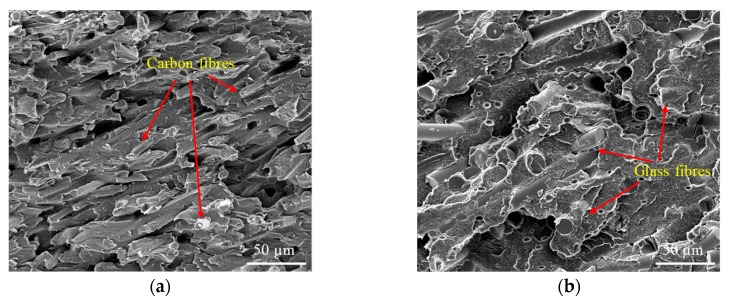

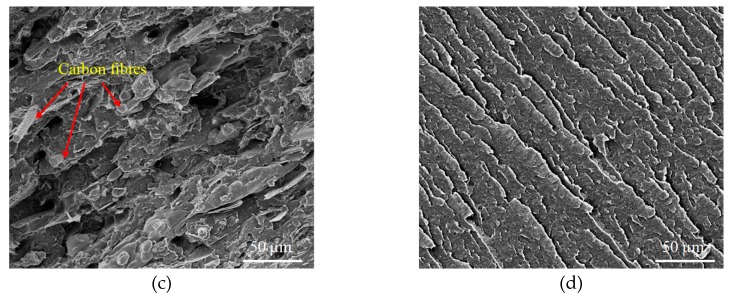
SEM images of fracture cross-section for polyetheretherketone (PEEK) and PEEK composites, (**a**) 30 wt % carbon fibre reinforced PEEK, (**b**) 30 wt % glass fibre reinforced PEEK, (**c**) each 10 wt % of PTFE, graphite and carbon fibre modified PEEK, (**d**) neat PEEK.

In this work, pure PEEK and three PEEK composites produced by injection moulding (Ensinger Ltd., Manchester, UK) were studied. The as-bought materials had the same shape and size (bar with outer diameter of 25 mm). The melting temperature was 334 °C from the manufacture’s data sheet. The three PEEK composites were: 30 wt % carbon fibre (~6 μm diameter) reinforced PEEK; 30 wt % glass fibre (~15 μm diameter) reinforced PEEK and each 10 wt % of PTFE, graphite and carbon fibre modified PEEK. Unfilled PEEK was tested for comparison. Figure 1 shows the SEM images of the fracture cross-section for PEEK and PEEK composites, in which how the reinforced fibres distribute and orientate in the matrix are indicated. These reinforced and unreinforced PEEKs were thereafter referred to Bush A, B, C and D respectively, listed in Table 1, including their mechanical and thermal properties.

**Figure 2:**
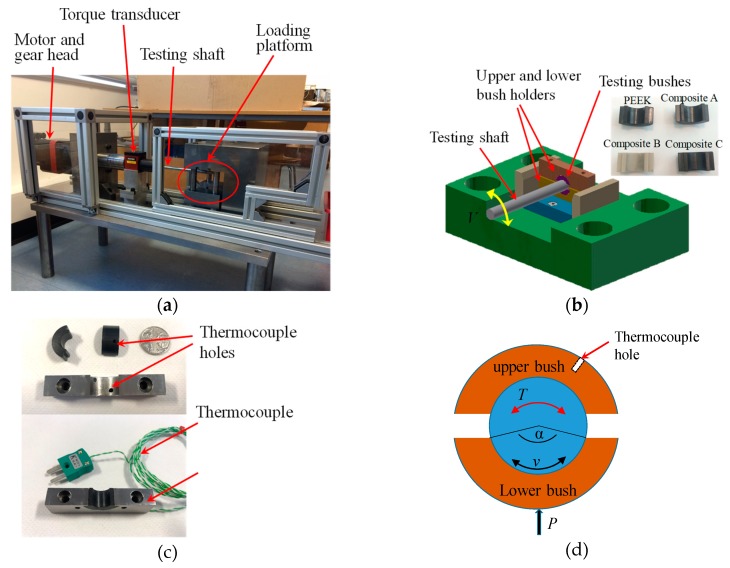
Pin joint test rig and bush specimen arrangement, (**a**) photo of the pin joint test rig; (**b**) loading platform with pin/bush assembly, bush halves located in two separate holders above and below the shaft, inset: bush specimens made of PEEK and PEEK composites; (**c**) thermocouple location; (**d**) contact geometry between shaft and bush halves subjected to normal load P applied from the lower bush, shaft oscillating speed v and required frictional torque T.

PEEK and PEEK composite bars were mechanically machined to bush halves for testing. The machining process involved turning the outer diameter to 15 and 20 mm, bored inner hole and precise reaming to the final inner diameter 10 mm, which left the roughness Ra = 0.7–1.1 µm for the bearing surface. Bushes were slit into halves to accommodate the loading design on the pin joint test rig, shown in Figure 2a. Figure 2b,c shows bush samples, bush holder and bush/pin contact configuration.

### 2.2. Wear Test

Wear tests were performed on a pin joint rig, shown in Figure 2a. To apply the normal load, the loading platform (shown in Figure 2b) with fitted lower bush holder was raised using an Enerpac RSM200 manual hydraulic cylinder, while the upper bush holder was kept static. The shaft was driven by an AKM42H (120 V) motor attached with a Micron XTRUE 160 planetary gearhead. In this study, the shaft performed an oscillating motion from −60° to +60° at a speed of 45 °/s (3.9 mm/s). A C-FW compression load cell (capacity of 100 kN) was located under the platform for measuring the normal load. A plunger dial indicator was attached to the loading platform to record its vertical displacement, which was the radial deformation occurring in the bush wall. A FUTEK FSH02059 torque transducer (200 Nm capacity, Irvine, CA, USA) was used to measure the frictional torque between the bush and shaft. The overall monitoring, recording and control of the rig was via a PC using a software program written in LabVIEW (National Instruments, Austin, TX, USA). More details of the test rig have been reported in [29].

A thermocouple hole was drilled in the bush wall, 1.2 and 2.5 mm deep against the inner surface for wall thickness 2.5 and 5 mm respectively, shown in Figure 2d. The temperature change in the wall material was measured using a K-type thermocouple. Figure 2c shows the assembly of bush, bush holder and thermocouple. A fresh bush pair was used for each test. Prior to test, bushes were cleaned with isopropanol in an ultrasonic bath. Bush wall thickness (WT) and mass were measured before and after each test. Mass loss (Δm) and wall thickness change were recorded for assessing the wear resistance.

A 300M steel shaft with a diameter of 10 mm and surface roughness Ra = 0.5 µm was adopted to contact with the bush specimen. It was cleaned with isopropanol prior to each test and reused. Tests were carried out without lubricant and at room temperature and humidity. Three repeats for each test were conducted using fresh bushes and a newly cleaned shaft.

Based on the contact geometry shown in Figure 2d, the nominal contact pressure between the rotating pin and the bush half, p, and the friction coefficient, μ, are calculated from the following equations,

(1) $$p=\frac{P}{2\mathrm{RLsin}\left(\alpha /2\right)}$$

(2) $$\mu =\frac{T}{2\mathrm{PR}}$$

where P is the normal load, 9 kN, R is the radius of the shaft, 10 mm, L is the contact width, and α is the arc angle of the bush halve. It should be noted that even though every effort has been made in sample preparation to reduce the variance between bush halves, it was impossible to have exactly identical samples. In this work, the contact mechanisms from the lower and upper bush halves were assumed to be the same, i.e., same friction force/torque occurred from each bush half.

Table 2 shows the testing conditions including the shaft and bush dimensions. Due to the varying reinforcements, differences in mechanical and tribological properties were expected. In order to fully understand their tribological capacity, varying test durations were applied. A defined radial deformation of the bush wall was used as an indicator to end the test. In testing, the reading from the plunger dial indicator was used to calculate the deformation in the bush wall. The test was manually stopped when the wall thickness change was 10% of its original thickness, which was defined as a failure in this study. The corresponding maximum articulating cycles were then compared among tested bushes. There was an exception for the Bush C (WT = 5 mm) caused by excessive lower deformation. In this case, the test was ended after 6 h running.

Friction coefficient and wear coefficient (referred to as mass loss) were used to analyse the contact mechanism between the shaft and bush. Wear was measured by mass loss, ∆m. Wear coefficient of the material W, in mm^3^/Nm, was calculated using the following equation,

(3) $$W=\frac{\Delta m}{\rho \mathrm{PS}}$$

where ρ is the density of the specimen listed in Table 1, P is the normal load, and S is the total sliding distance.

During the test, frictional heating occurred [30] at the contact between the shaft and bush halves due to combined normal and tangential loading. As polymers are more sensitive to mechanical stresses and temperature [31], it is necessary to take into account frictional energy in the investigation of the friction and wear properties. For the current contact configuration, the frictional energy equals the work required to enable the shaft to rotate inside the bush halves. It is calculated by the following equation,

(4) $$E=\mathrm{Pv}\int_{t_{s}}^{t_{e}}\mu \left(t\right)\mathrm{dt}$$

where v is the sliding speed, 3.9 mm/s, μ(t) is the coefficient of friction (CoF), the shaft starts articulating at t_s_ and ends at t_e_.

Specific wear energy [23] that combines friction coefficient and wear was used to assess the friction and wear properties. It is the ratio of the frictional work divided by the bush mass loss in the wear process, shown as the following equation,

(5) $$E_{w}=\frac{E}{\Delta m}=\frac{\mathrm{Pv}\int_{t_{s}}^{t_{e}}\mu \left(t\right)\mathrm{dt}}{\Delta m}$$

### 2.3. Characterization

In this study, an Inspect F FEG-SEM (FEI, Eindhoven, Netherlands) was used to characterize worn surfaces of the tested samples. Wear debris were assessed using an Alicona InfiniteFocusSL microscope (Alicona Imaging GmbH, Graz, Austria). The contact zone on the shaft after each test was examined by using an optical microscope (Zeiss Optical Microscope, Cambridge, UK). The bush mass was measured using a Sartorius Electronic Analytical Balance BP210D (accuracy 0.01 mg).

## 3. Results and Discussions

### 3.1. Friction and Wear

**Figure 3:**
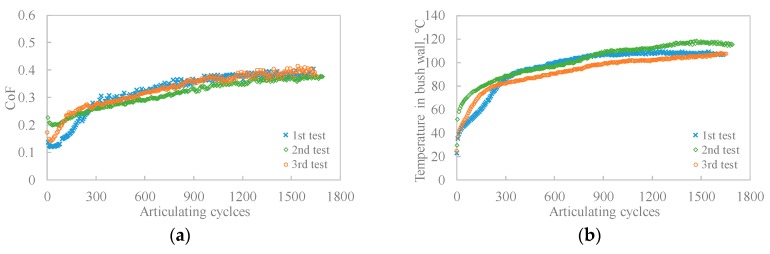
Three repeat wear tests for Bush A: (**a**) CoF varying with articulating cycles; (**b**) temperature rise in the bush wall varying with articulating cycles.

Figure 3 presents three repeats of the wear test for Bush A, characterised by CoF and temperature in the bush wall. It can be seen that good repeatability was seen among the three repeats. The small difference of CoF and temperature curves may arise from the variance of specimen surface texture and roughness produced in the process of mechanical machining.

**Figure 4:**
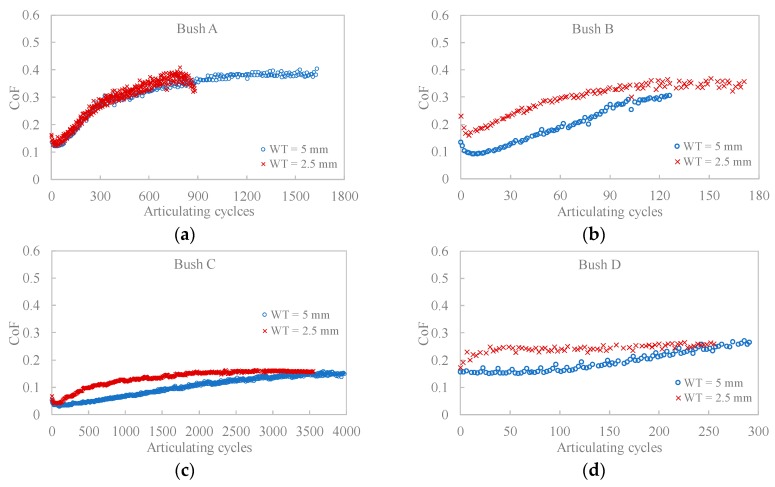
Typical evolution of CoF and recorded temperature varying with articulating cycles for (**a**) Bush A; (**b**) Bush B; (**c**) Bush C; (**d**) Bush D.

**Figure 5:**
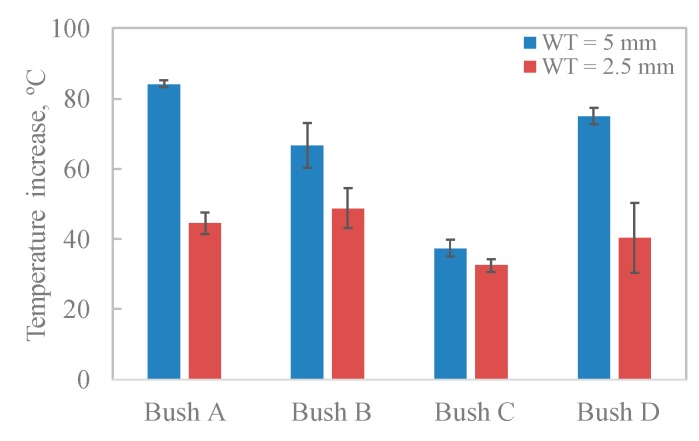
Temperature increase in the middle of the bush wall thickness.

The comparison of CoF and temperature increase among four composite bushes are shown in Figure 4 and Figure 5. The tests for Bush A, B and D were stopped when the bush wall thickness reached a 10% change compared with the original size. Overall, the CoF increased over the testing period for all bushes while the composite C presented the lowest CoF values and temperature increase. This indicated that the incorporation of PTFE, graphite and carbon fibre significantly reduced both the friction and temperature rise.

Comparing the two bush wall thicknesses, the thinner ones had slightly higher CoF and lower temperature increase. As they were subjected to the same testing conditions, the difference in CoF could only be caused by the energy dissipation efficiency. In other words, the contact temperature played as an influential factor for the contact mechanism. Apparently, thinner wall bushes reduced the accumulation of the friction heat by dissipating heat to the adjacent metal parts. While for thick wall bushes, the increase in contact temperature decreases the stiffness of the matrix, the shear strength, and therefore resulted in a lower CoF [32].

For the first 300 cycles in Figure 4a,d, the carbon fibre reinforcement in Bush A did not seem to reduce the friction as unfilled PEEK shows a constant and relatively lower CoF. Compared with Bush B (glass fibre reinforced) and Bush D (unfilled PEEK), Bush A did show improved bearing capacity (higher articulating cycles), which is in agreement with the findings of [1].

After each test, the bush wall thickness was measured to determine maximum wall reduction, as shown in Table 3. Under the same load, the thinner bushes presented more deformation indicating lower load bearing capacity. For Bush A, the highest radial deformation, 14.09% reduction, was observed at WT = 2.5 mm. Unsurprisingly, Bush C showed the lowest deformation for both wall sizes. During the wear test, there was no wear debris observed for Bush B and D. The bush mass loss was also too low to be measured. The wear coefficients for Bushes A and C were calculated from their mass loss and are shown in Table 3. Both CoF and wear loss for Bush A were found to be significantly higher than that of Bush C. In other words, Bush C exhibited notably superior bearing properties among the four tested composites. From this study, it is clear that no correlation between mass loss and bearing capacity can be concluded. The friction coefficient and wear loss did not provide enough information to disclose the contact mechanism either. As there was no wear loss occurred on Bush B and D, in order to compare the contact mechanism among the tested bushes, it is necessary to study the interface at a microscopic scale.

### 3.2. Wear Debris and Worn Surfaces

**Figure 6:**
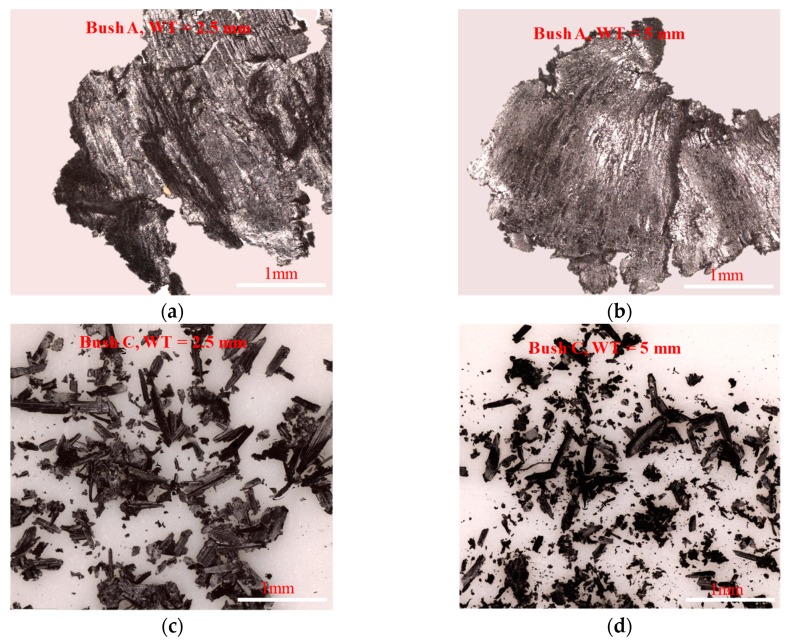
Typical debris observed from the wear test for (**a**) and (**b**) Bush A; (**c**) and (**d**) Bush C for wall thickness 2.5 and 5 mm, respectively. No wear debris observed from Bush B (glass fibre reinforced PEEK) and Bush D (unfilled PEEK).

In order to understand the wear mechanism, wear debris from Bush A and C were collected and assessed using the Alicona InfiniteFocusSL, shown in Figure 6. No wear debris were observed from Bush B (glass fibre reinforced) and D (unfilled PEEK) from the bush wear test. For carbon fibre reinforced Bush A, large fragments of debris, up to 3–5 mm in length, were observed, while the 2.5 mm wall bush produced similar but thicker flakes, shown in Figure 6a,b. The highest wear loss and wear coefficient were presented by Bush A when WT = 2.5 mm. Figure 6c,d shows much finer wear debris from Bush C. Slightly coarse wear particles were found for thicker wall bush. The presence of graphite and PTFE in the matrix reduced the formation of larger debris chips.

**Figure 7:**
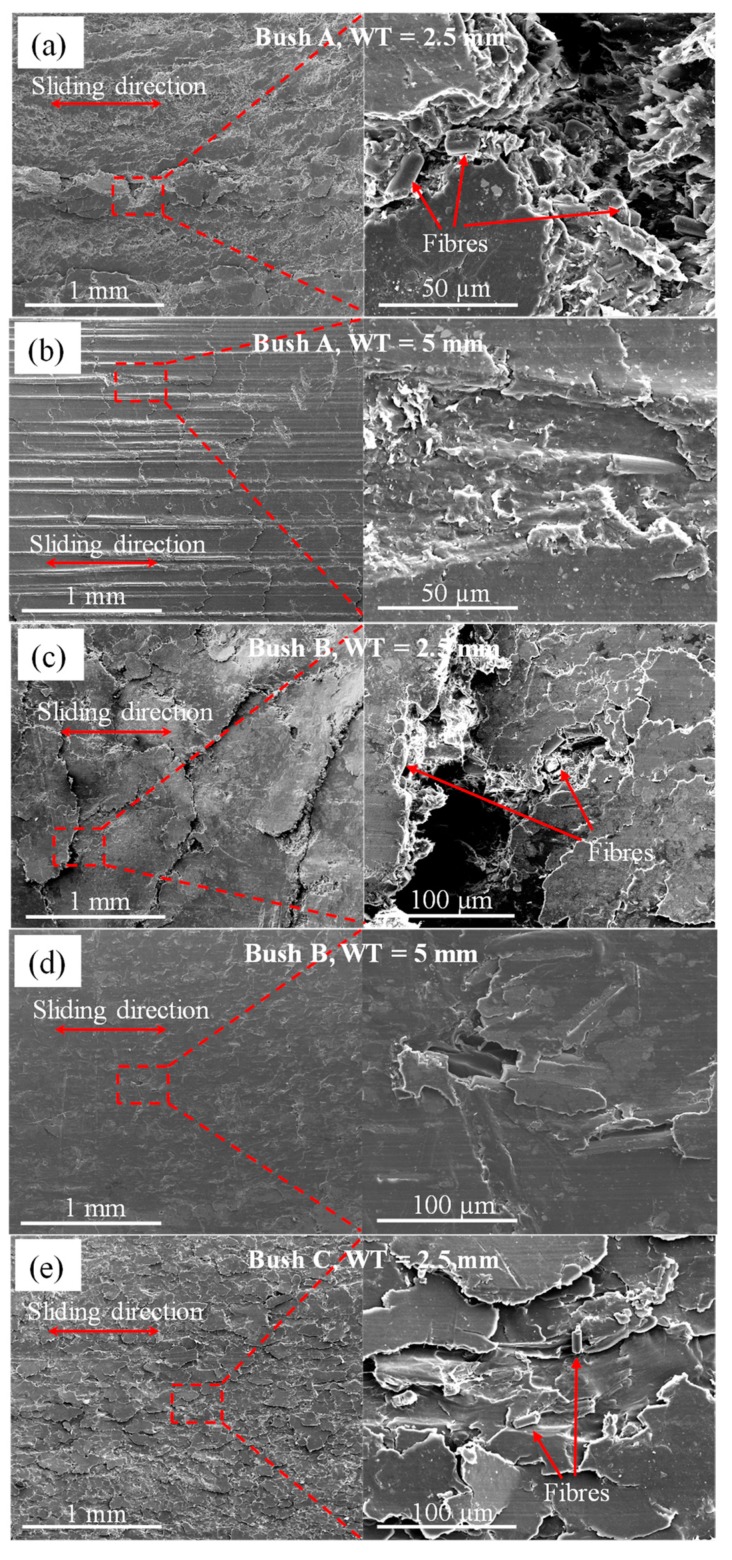

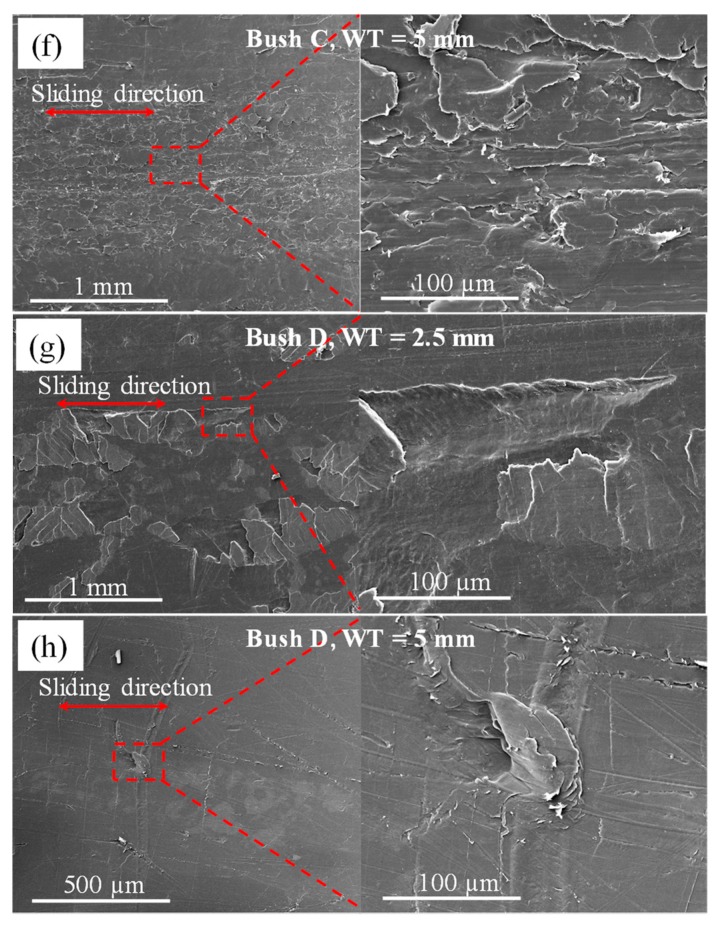
SEM images of worn bush surfaces, (**a**) and (**b**) Bush A; (**c**) and (**d**) Bush B; (**e**) and (**f**) Bush C; (**g**) and (**h**) Bush D for wall thickness of 2.5 and 5 mm.

Figure 7 gives lower and higher magnification SEM images of the top-view worn surfaces from tested bushes. It is clear that under the combined action of compression, shearing and frictional heating, PEEK and PEEK composites displayed diverse patterns on the surface layer, caused by different wear mechanisms. Due to repeated stressing, cracks were produced at the surface and/or just sub-surface in the composite. These cracks gradually grew and joined each other until wear debris, including spalls, were detached after a certain number of stressing cycles. Therefore, adhesion and fatigue were the main wear mechanisms occurring at the interfaces. Bushes with thinner walls showed patches of overlapping platelets on the surface, demonstrating a severe deformation and shearing of the surface materials.

Bush B showed the overall worst case, with large blocky particles over 1 mm in length. For fibre reinforced matrixes, shown in the higher magnification images in Figure 7a,c,e, fibres were pulled out, broken and crushed, either exposed on the surface or pressed in the deformed layer. Through block-on-ring test, Zhang et al. found that carbon fibre thinning (fibre wear) dominate the wear mechanism at low pressure of 1MPa [33]. This phenomenon was not observed on tested bushes. It implies that under higher contact pressure (93 MPa), the contact mechanism mainly fell in severe deformation and tearing of surface material. Glass fibres or carbon fibres were broken into short pieces, remaining the same diameter, rather than gradually being thinned by shear stress caused fatigue.

For the thicker wall bushes, a smoother surface layer (Figure 7b,d,f,h) was observed under the same loading and sliding conditions. Continuous micro ploughing along the sliding direction associated with platelet patches was exhibited by Bush A at WT = 5mm. The wear mechanism falls into a combination of abrasion and adhesion. These slightly ‘smoother’ surface topographies agreed with higher articulating cycles in Figure 4, inferring greater bearing capacities for thick wall bushes. Compared with other bushes, Bush C showed smoother surfaces without gaps between platelet patches. This was due to the existence of self-lubricating agents, graphite and PTFE, reducing the shear stress on the interface. Obvious matrix shear failure was rarely observed for Bush D (unfilled PEEK) while the worn surface showed some patches detached on the surface. Plastic flow was observed at WT = 5mm. Bush B and D showed comparable articulating cycles and radial deformation which were much worse than Bush A and C. Glass fibres did not bring any enhancement to the mechanical strength or friction and wear resistance of the matrix.

### 3.3. Cross Section of Worn Surfaces

**Figure 8:**
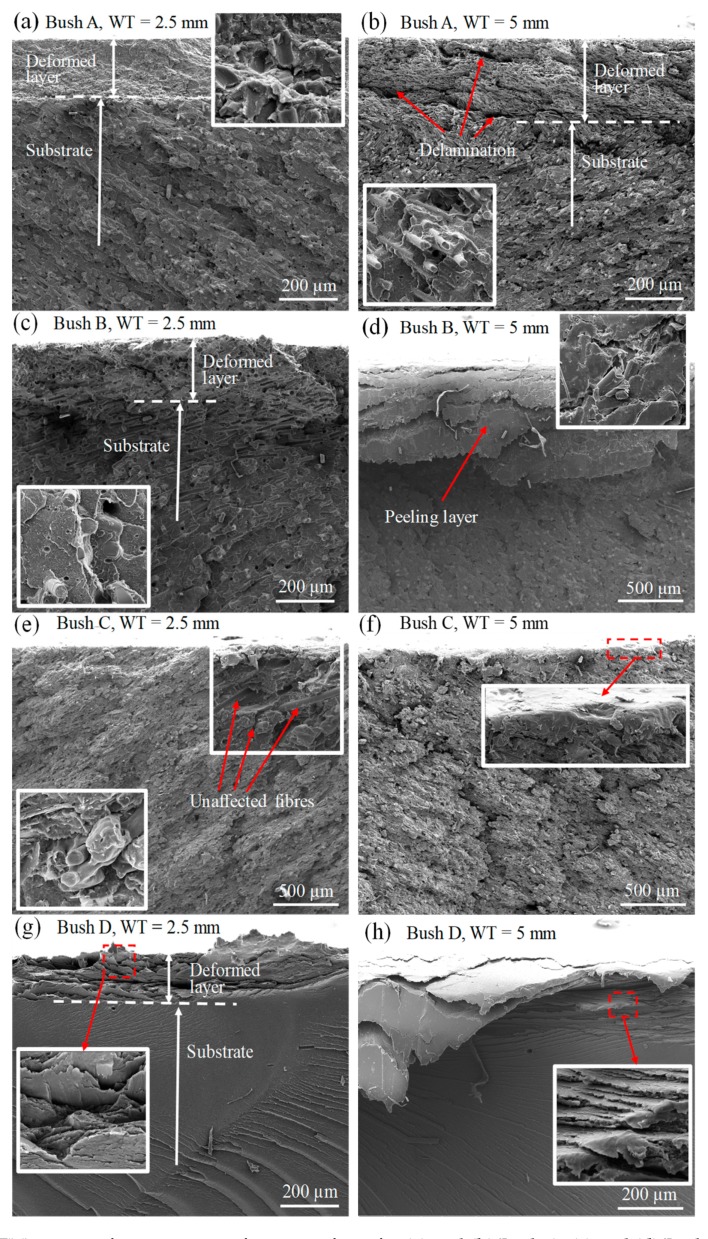
SEM images of cross section of worn surfaces for (**a**) and (**b**) Bush A, (**c**) and (**d**) Bush B, (**e**) and (**f**) Bush C, and (**g**) and (**h**) Bush D for wall thickness of 2.5 and 5 mm, respectively.

Worn surface morphology is useful in the analysis of friction and wear behaviour, while subsurface material change provides important information regarding the bulk properties such as load carrying capacity, resistance to compression, cracking and fatigue. After testing, bush halves were quenched in liquid nitrogen and fractured to expose the cross-section. Figure 8 shows fractography for tested bushes after cyclic loading in normal and tangential directions. The direction of sliding is into the page. An extensively deformed subsurface layer was observed for all bushes except Bush C. For example, Figure 8a for the 2.5 mm thick Bush A shows a 200 μm thick layer composed of deformed material on top of the substrate matrix. The surface layer material appears compressed by the high normal load. The inset image shows the deformed matrix with broken carbon fibres and the PEEK. The surface layer in Figure 8b for WT = 5 mm is slightly thicker, around 270 μm, but less compressed as delaminated sub-layers can be seen. The inset image in Figure 8b shows an unmodified substrate matrix. It is reasonable to conclude that the deformation occurring in the surface layer is an attribute of the wall thickness reduction. Chen et al. claimed that the exposed carbon fibre on the sliding surface carried most of the normal load and therefore improved the matrix load bearing capacity [1]. While in this study, this thick surface layer stacked on top of the substrate matrix was presumed to support the normal load and dissipate frictional heat into bulk material underneath.

Bush B showed a similar deformed depth after 171 articulating cycles (Figure 8c) compared with Bush A of 882 articulating cycles (Figure 8a). A layer was detached from the substrate matrix, shown in Figure 8d. Again, in this layer, glass fibres were found to be fractured, crushed and blended in the matrix shown in the inset. Cross sections of the unfilled PEEK bush are shown in Figure 8g,h. Plastic flow has occurred shown by the inset in Figure 8h.

Unsurprisingly, the best performance was shown by the Bush C (Figure 8e,f). Only a very thin surface layer was affected by compression and shearing. In the sliding contact under normal load, strain occurs both in the normal and tangential directions [33,34]. For the reinforced matrix, normal and shear stresses transferred at the interface between reinforcements and the matrix material. Due to the very low surface energy of graphite and PTFE, the shear stress is low leading to a low CoF. As the carbon fibres were dispersed uniformly in the matrix with varying orientations, the normal stress was therefore supported effectively by high modulus carbon fibres in varying directions [33].

### 3.4. Worn Surface on Shafts

**Figure 9:**
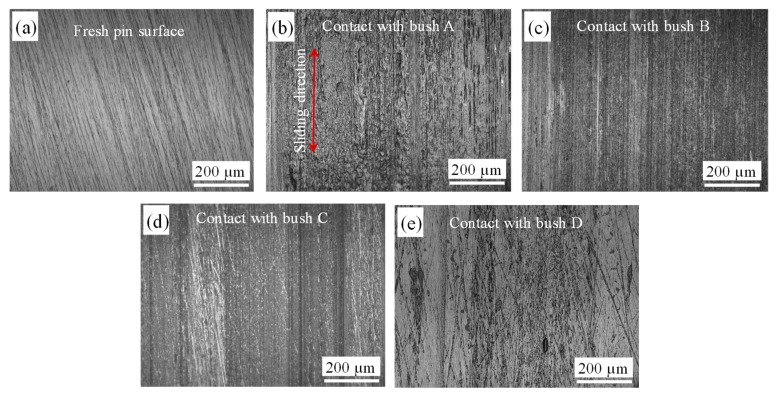
Microscopic images of the shaft surfaces, (**a**) fresh surface; (**b**) contacted with Bush A; (**c**) contacted with Bush B; (**d**) contacted with Bush C; (**e**) contacted with Bush D.

Figure 9 shows microscopic images of the pin surface before and after contacting with bush samples (WT = 5 mm). The sliding direction is marked on Figure 9b. Compared with the fresh surface (Figure 9a), a continuous thick layer of transferred material was observed for the Bush A (Figure 9b). It is clear that material transfer from bush surface has occurred. Weakening and debonding between carbon fibres and the matrix were expected due to the shearing and compression. In sliding, when the friction force is greater than the adhesive interaction between polymer matrix and reinforcements, the asperities of the composite material can be removed to form a transfer layer on the counterface [35]. Bely et al. [36] reported that the transfer of polymer is the most important characteristic of adhesive wear in polymers. The adhesion process is normally associated with other wear types (fatigue, abrasion and so on) [35].

There was no visible wear debris accumulated next to the sample for Bushes B and D after testing. However, due to the extreme hard of glass fibres in the Bush B, fine furrows were observed along the sliding direction, shown in Figure 9c. Without contacting with hard glass fibres, the shaft surface presented limited wear and material transfer (Figure 9e). Even a thin layer of bush material was deposited on the pin surface, no measurable mass loss was produced for neither Bush B or D. This indicated that only the top asperity layer was removed from the bush surfaces while the bulk matrix underwent a plastic deformation.

### 3.5. Friction Energy

Over the testing duration, the friction energy that is transformed as a consequence of frictional contact is dissipated and converted to heat, vibration, material deformation or stored in the tribo-system. For sliding between the shaft and bush, the frictional energy and specific frictional energy were calculated from Equations (4) and (5), respectively (see Table 4). The table includes the overall temperature increase (temperature difference between the start and end of each test). It can be seen that bush A and C show significantly higher frictional energy compared to Bush B and D. This is because the testing durations (articulating cycles) were much longer than that of Bush B and D. Low thermal conductivity usually leads to the accumulation of the frictional heat and therefore reduces the bearing capability of composites [37]. This explains the performance of Bush B and D, due to their low thermal conductivities, they showed higher temperature increase and lower articulating cycles to failure. This is also evidenced by the formation of a peeling layer shown in Figure 8d,h. The multilayers immediately beneath the wear track in Figure 8h show the plastic deformation due to the combined action of reciprocating shearing and thermal softening. However, Bush A and C have similar thermal conductivities, 0.92 and 0.82 Wm^−1^°C^−1^, respectively; the articulating cycles of Bush C are more than twice that of Bush A. In this case, CoF played an important role in the temperature increase. In other words, in order to achieve good performance of the bearing bush, both low friction coefficient and high thermal conductivity are required [38].

**Figure 10:**
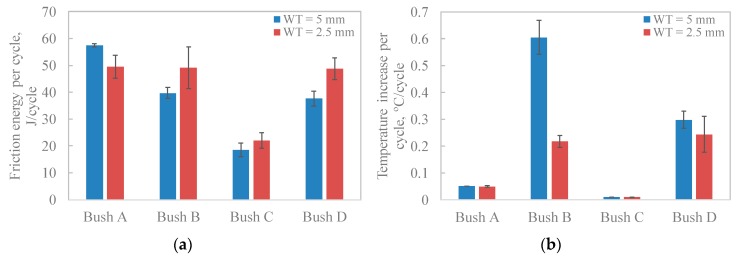
Averaged friction energy and temperature increase per cycle for tested bushes, (**a**) friction energy per cycle; (**b**) temperature increase per cycle.

Due to a significant difference in articulating cycles for each bush test, it is better to compare bushes using friction energy per cycle (Figure 10a) and temperature rise per cycle (Figure 10b). It can be seen that Bush C produced the least friction energy per cycle due to the lowest CoF. Less frictional heat produced at the interface leads to lower temperature increase in the material bulk, shown in Figure 10b. The graphite lamellar structure reduced CoF and hence the heat generation. In addition, during material deformation, PTFE helped to store much of the work, which was used in crystallographic and amorphous chain rearrangement, resulting in less sample heating [39]. Therefore, the thermal softening in Bush C was limited. This agreed with the microscopic morphology displayed in Figure 8e,f where the lest deformation of the material was observed.

## 4. Conclusions

A comparative study of PEEK composite bushes for use in articulating revolute pin joints has been conducted. The friction, wear, friction energy and temperature rise have been studied. The friction and wear mechanisms were assessed by studying the microscopic worn surfaces and deformation layer beneath. The thermal accumulation and dissipation were studied to improve the understanding of the tribological performance for PEEK composite used as bearing bushes.

Due to low thermal conductivity, unfilled PEEK and glass fibre reinforced PEEK presented much lower articulating cycles to failure than that of graphite, PTFE and carbon fibre filled PEEK. The load bearing capacity of the composite is much higher than that of the matrix, and thus, any sub-surface fracture and yielding are diminished due to the presence of the hard and strong reinforcements. Presence of graphite and PTFE in the PEEK matrix not only reduced shear force at the interface but also minimised the temperature increase in the bulk material. In addition, the wear resistance was significantly improved. The wear coefficient of Bush C was found to be 0.13 × 10^−6^ mm^3^/Nm compared with 4.33 × 10^−6^ mm^3^/Nm for Bush A.

Bushes made of PEEK composite formulated with PTFE, graphite and carbon fibre exhibit low friction, self-lubricating, low temperature rise, and therefore present superior bearing properties, including enhanced bearing life and reducing energy consumption in machinery. The findings facilitate the application of this PEEK composite used as self-lubricating bearing bushes.

## Figures and Tables

**Table 1:** Composition, mechanical and thermal properties of PEEK and PEEK composites [28].

Specimen	PEEK Composite Reinforcements	Density, g/cm^3^	Elastic Modulus, GPa	Compression Strength @ 10% Strain, MPa	Rockwell Hardness, M Scale	Elongation at Break @22.8 °C, %	Thermal Conductivity, Wm^−1^°C^−1^
Bush A	30 wt % carbon fibre	1.41	6.34	165	107	7	0.92
Bush B	30 wt % glass fibre	1.53	6.89	172	103	2.2	0.3
Bush C	10 wt % each, carbon fibre, graphite, PTFE	1.46	5.52	114	95	2.5	0.82
Bush D	None	1.31	4.48	121	99	40	0.29

**Table 2:** Oscillating test conditions.

Nominal Contact Pressure	Articulating Displacement	Articulating Speed	Pin Radius	Bush Arc Angle	Bush Width	Bush Wall Thickness	Oscillating Cycles
p = 93 MPa	−60° to +60°	45 °/s (3.9 mm/s)	R = 5 mm	120°	L = 10 mm	WT = 2.5/5 mm	Vary

**Table 3:** Maximum radial change of the bush wall and wear coefficient for tested bushes.

	Bush A		Bush C	
WT, mm	2.5	5	2.5	5
Wear coefficient, ×10^−6^ mm^3^/Nm	4.33 ± 0.78	3.76 ± 0.65	0.73 ± 0.04	0.13 ± 0.04

**Table 4:** Frictional energy for tested bushes.

	Bush A		Bush B		Bush C		Bush D	
WT, mm	2.5	5	2.5	5	2.5	5	2.5	5
Friction energy, ×10^4^ J	4.46 ± 0.48	9.53 ± 0.01	1.08 ± 0.11	0.44 ± 0.01	7.52 ± 1.59	7.15 ± 1.12	0.84 ± 0.16	0.97 ± 0.13
Specific wear energy, ×10^4^ J/mg	0.29 ± 0.06	0.46 ± 0.09	-	-	0.79 ± 0.07	4.36 ± 0.64	-	-
Temperature rise, °C	44.5 ± 3.06	84.16 ± 0.9	48.74 ± 5.58	66.54 ± 6.37	32.47 ± 1.8	37.4 ± 2.31	40.33 ± 10.03	75.02 ± 2.4
